# High-Speed THz Time-of-Flight Imaging with Reflective Optics

**DOI:** 10.3390/s23020873

**Published:** 2023-01-12

**Authors:** Hoseong Yoo, Jangsun Kim, Yeong Hwan Ahn

**Affiliations:** 1 Department of Physics and Department of Energy Systems Research, Ajou University, Suwon 16499, Republic of Korea; 2 Panoptics Corp., Seongnam 13516, Republic of Korea

**Keywords:** terahertz imaging, nondestructive testing, time-of-flight

## Abstract

In this study, we develop a 3D THz time-of-flight (TOF) imaging technique by using reflective optics to preserve the high-frequency components from a THz antenna. We use an Fe:InGaAs/InAlAs emitter containing relatively high-frequency components. THz-TOF imaging with asynchronous optical sampling (ASOPS) enables the rapid scanning of 100 Hz/scan with a time delay span of 100 ps. We characterize the transverse resolution using knife edge tests for a focal length of 5; the Rayleigh resolution has been measured at 1.0 mm at the focal plane. Conversely, the longitudinal resolution is determined by the temporal pulse width, confirmed with various gap structures enclosed by a quartz substrate. The phase analysis reveals that reflected waves from the top interface exhibit a phase shift when the gap is filled by high-indexed materials such as water but shows in-phase behavior when it is filled with air and low-indexed material. Our imaging tool was effective for inspecting the packaged chip with high lateral and longitudinal resolution. Importantly, the phase information in 2D and 3D images is shown to be a powerful tool in identifying the defect—in particular, delamination in the chip—which tends to be detrimental to the packaged chip’s stability.

## 1. Introduction

Terahertz (THz) spectroscopy has emerged as a promising tool in a wide range of applications, such as material characterization, safety inspection, biomedical diagnosis, device inspection, telecommunication, and sensors [1,2,3,4,5,6,7,8]. In particular, the THz technique is desirable for the nondestructive testing (NDT) of the internal structures of objects. This is possible because THz waves are frequently transparent to the enclosures when they are made of nonconductive materials [9,10,11,12,13,14,15,16,17,18,19,20]. The recent technological advances in novel THz sources and detectors have increased the feasibility of effective THz imaging systems for practical application—for example, to identify ithe size, shape, and location of conductive objects and defects in insulating materials [21,22,23,24,25,26,27,28,29]. In addition, THz imaging, which is capable of 3D mapping on objects, has attracted particular interest because it allows users to locate their vertical as well as lateral positions. Coherent THz tomography, based on frequency-modulated CW (FMCW) methods, has been widely investigated using tunable or ensemble sources. However, it is limited in terms of longitudinal resolution [30,31,32]. In contrast, the time-of-flight methods based on time-domain spectroscopy (TDS) deliver better resolution in general, without using frequency sweeping methods. Here, the longitudinal resolution is determined by the temporal width of the pulsed laser source, enabling us to readily achieve 150 μm when the pulse width reaches 1 ps. Conversely, the relatively slow measurement time has limited the use of THz-TOF imaging for practical applications.

Recently, rapid THz-TOF imaging methods have been introduced for the inspection of packaged integrated circuits and defects in insulating materials [33,34]. An imaging rate of 100–200 Hz/pixels has been achieved based on the THz–TDS technique where fast time-delay scanning methods have been implemented. The optical sampling by cavity tuning (OSCAT) system delivers a rapid time delay with a full range of 50 ps, whereby a single pulsed fiber source is split into two pulses used for the THz antenna and receiver, respectively [35,36]. Conversely, THz–TDS systems with asynchronous optical sampling (ASOPS) use two synchronized fiber lasers and deliver a time delay reaching 10 ns, which is determined by the laser repetition rate of 100 MHz [37,38]. The acquisition rate is determined by the repetition rate difference of the two fiber lasers that are selected to be approximately 100–500 Hz, depending on the signal-to-noise ratio. THz spectroscopy and imaging with the electronically controlled optical sampling (ECOPS) technique has been introduced using two independent lasers; however, fast imaging with galvanometer scanning has not been demonstrated yet [39,40,41].

For fast imaging with a speed of more than 100 Hz for each A-scan, it is important to incorporate galvano scanning methods in front of the scan lens. Recently, a THz antenna with a frequency range larger than 1 THz based on iron-doped InGaAs/InAlAs materials has become available [42]. Conventional THz imaging systems based on CW and pulsed sources have been implemented with refractive optics (transmission lenses). They were constructed of materials that were transparent against THz waves—for example, high resistivity float zone silicon (HRFZ-Si), quartz, fused silica (FS), and polymers [43]. However, there is noticeable attenuation in the high-frequency range larger than 1 THz. In addition, there is an inevitable reflection loss that is determined by the refractive index of the materials. Therefore, to optimize the new THz emitters both in terms of resolution and transmission efficiency, it is essential to adopt reflective optics instead of refractive optics, which will enable virtually loss-free configuration, especially in the high-frequency range. Conversely, the use of reflective optics in fast THz imaging systems has not yet been demonstrated.

In this study, we developed rapid THz-TOF imaging results consisting of reflective optics, in which a novel THz antenna delivers a better spatial resolution and enhanced signal-to-noise ratio. Using a focused lens of 50 mm, we characterized the imaging system by measuring the resolution in the transverse and longitudinal directions. We also introduced a novel phase mapping technique that is efficient in identifying delamination defects in the packaged semiconducting chips.

## 2. Experimental Setup

**Figure 1:**
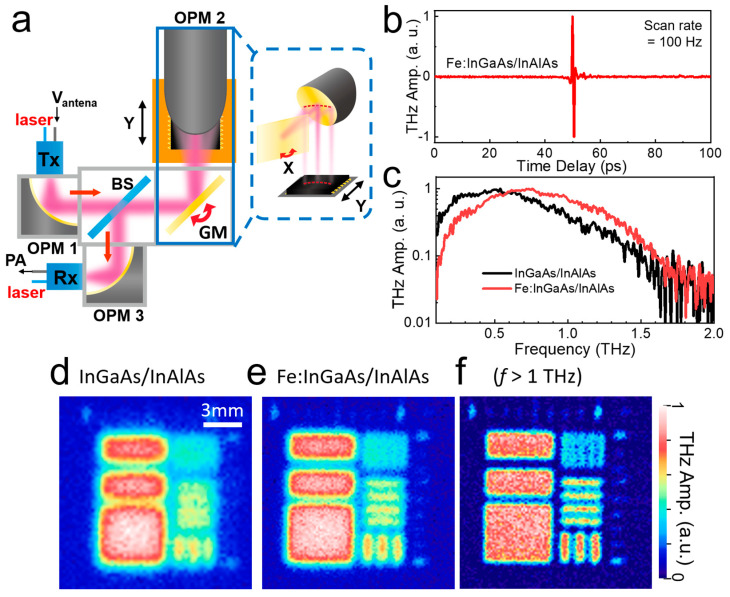
(**a**) Schematic illustration of the THz-TOF imaging setup with all-reflective optics. (**b**) A representative THz reflection amplitude as a function of time delay (A-scan). (**c**) THz reflection spectra for both antenna types (**c**–**e**) Time-integrated reflection amplitude images on a test pattern (C-scan) with old antenna (**d**), new antenna (**e**), and high-frequency filtered image with the new antenna (**f**). GM: galvanometer, BS: beamsplitter, OPM: off-axis parabolic mirror, PA: current preamplifier.

A schematic of the THz-TOF imaging equipment is illustrated in Figure 1a. For the rapid THz–TDS system, we used a commercialized ASOPS instrument (TERA-ASOPS, MenloSystems GmbH), consisting of two-independent femtosecond fiber lasers with a wavelength of 1560 nm, operating with a repetition rate of 100 MHz. The ASOPS system allows a full time-delay range of 10 ns with a rate of 100–500 Hz/pixel, which we fixed at 100 Hz/pixel throughout the experiments. We recorded the data with the time span of 100 ps (out of the full delay scan range of 10 ns), as shown in Figure 1b, because the transverse resolution is limited by the depth of focus, as will be discussed later. THz-TOF with ASOPS has a significant advantage in terms of large-depth profiling, whereby it remains restricted by the focal depth of the scan imaging system. In particular, we incorporated a novel THz antenna with a large bandwidth based on the iron-doped InGaAs/InAlAs material, which delivers a large amount of high-frequency components, as shown in Figure 1c. We note that there is a hemispherical lens attached to the commercialized antenna (for both emitter and detector), which is used to guide the THz beams with a divergence angle of 29°. High-frequency components are critical for obtaining images with high resolutions. In general, conventional refractive optics in the image scanning system are typically made of HRFZ-Si or polymethylpentene (TPX) and attenuate a large portion of the high-frequency components. In the TOF imaging system, the THz pulse passes through the lenses four times, including transmission through a scan lens (back and forth) in front of the sample and through two lenses in front of the emitter and detector.

Here, we developed THz imaging systems based on pure reflective optics (Figure 1a), except for the hemispherical lens attached to the commercialized antenna. We used three off-axis parabolic mirrors (OPMs) and a one-axis galvano scanner. An OPM with a diameter of 25 mm and a focal length of 50 mm was used in front of the THz emitter (denoted by Tx) to collimate the THz beams from the emitter. The other OPM, with a diameter of 50 mm and a focal length of 50 mm, was used to focus the THz beam onto the samples. To simplify the imaging systems while obtaining a speed that is limited by the ASOPS THz–TDS system, we combined fast galvano scanning for the *x*-axis and slow stage scanning for the *y*-axis. The reflected THz signal was collected using a 50:50 beam splitter (Tydex Inc. St. Peterburg, Russia), which was focused onto the THz receiving antenna (denoted by Rx) by using another OPA. The same specification used for the emitter was used for the detector. We note that distortion in the raster-scanned image occurs particularly when the scan position is far from the center. This is because conventional OPMs are designed to focus the collimated beams, not to raster-scan with the galvano scanning system. Correction of the image distortion originating from the imperfect *f*-*θ* scan lens and galvano scanning systems has been widely investigated [44]. It is relatively simple in our case, because the correction is needed along one direction, because we combined the sample stage scanning for another axis (*y*-axis). The image distortion differs, depending on the scan direction. In this case, we chose to scan along the horizontal line (inset, Figure 1a). Distortion in the image can be corrected post-measurement or in situ by slightly adjusting the *y*-position of the sample stage along the curved line. In addition, the signal amplitudes were normalized by reference signals—in other words, by the position-dependent reflection signal from the gold plate.

The current signal from the antenna was amplified using a fast current preamplifier (DLPCA-100; FEMTO Messtechnik GmbH) and digitized with a data acquisition card (ATS9462, Alazar Tech. Inc., Pointe-Claire, QC, Canada) with a rate of 10 MHz. This allowed us to obtain a time-delay step of 0.1 ps. The collected data contained phase-sensitive THz amplitudes as a function of the 3D parameters, such as the time delay, *x*-axis, and *y*-axis. We applied the point average to improve the signal-to-noise ratio, leading to a measurement rate of 5–10 Hz/pixel, while the system delivered 100 Hz/pixel. We used a delay-scan range of Δ*T* = 100 ps, which corresponds to a vertical range of 15 mm in the case of free-space propagation. Here, the time delay can be converted into depth information, in which 1 ps corresponds to 0.15/*n* mm in the reflection geometry, where *n* is the refractive index of the materials [33,34]. The images were recorded as a single binary file, which can be reconstructed using home-built analyzing software. The phase-sensitive THz amplitudes can be converted into the envelope signal by using the Hilbert transformation—that is, from the complex THz signal E˜THz=Re(E˜THz)+iH[Re(E˜THz)], where *H* is the Hilbert transformation [45].

Shown in Figure 1d,e are C-scan images for time-integrated reflection amplitudes on the test alignment pattern (Au pattern on Si substrate), when we used old and new antennae, respectively. Clearly, the spatial resolution improved with the new antenna, having more high-frequency components. The resolution improves even further if we filter the high-frequency component (>1 THz), as shown in Figure 1f. Obviously, it is essential for practical applications to develop THz sources and detectors having higher-frequency components while delivering fast scan rates. In addition, the use of reflective optics allows us to reduce the reflection losses originating from the finite refractive index of the lens material.

## 3. Results

### 3.1. Measurements of Transverse Resolution

**Figure 2:**
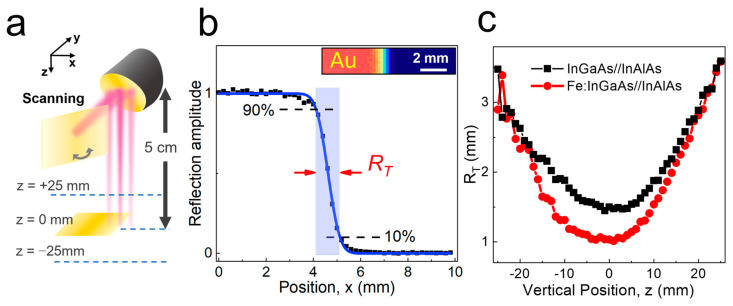
(**a**) Schematic illustration for transverse resolution measurement for different *z*-locations with a knife edge. (**b**) THz signal (black squares) as a function of position (*x*) when the metal plate is located at *z* = 0 mm. The blue solid line is the fitted curve with the error function. *R*_T_ denotes the transverse resolution defined with 10–90% transition of THz amplitudes. (inset) Time-integrated C-scan image of the reflected wave envelope from the knife edge. Au indicates gold surface. (**c**) The transverse resolution (*R*_T_) as a function of the vertical position (*z*) of the metal plate, extracted from the fitting result in (**c**) for both InGaAs/InAlAs and Fe:InGaAs/InAlAs antennas.

We first characterized the transverse resolution of our imaging system by comparing it with the old emitter. As shown in Figure 2a, we used a metal plate with a knife edge to measure the transverse resolution of the system. We obtained THz images for different heights (*z*) of the metallic plate, from *z* = −25 to *z* = +25 mm, where *z* = 0 indicates the location of the focus plane, which was positioned 5 cm below the lens. Figure 2b illustrates the line profile of the reflected amplitude signal as a function of the lateral position (*x*), extracted from the spectrally integrated C-scan images shown in the inset. Here, the vertical location was fixed at *z* = 0 mm (Figure 2a). The reflection signal was high when it was reflected by the metal plane (*x* < 4 mm), whereas it was suppressed when the focused beam was positioned outside of the metal (on the right side of the knife edge).

The line profile was fitted with the error function to obtain the transverse resolution of the focused THz beams. We determined the transverse Rayleigh resolution (*R*_T_) using the knife-edge 10–90% transition in THz amplitude in Figure 2b. By fitting the curve, the *R*_T_ of the reflected THz amplitude was measured at 1.0 mm, which corresponds to the Rayleigh half-pitch resolution of 0.5 mm [46,47]. The extracted *R*_T_ as a function of the vertical position (*z*) of the metal plane is plotted for both antenna types (Figure 2c). The minimum resolution was achieved at approximately *z* = 0 mm and it increased when the metal plane was away from the focused plane. For example, it increased up to *R*_T_ = 2.3 mm for *z* = −20 mm. In contrast, we could readily achieve *R*_T_ in the range of 1.0–1.1 mm within a depth range of 10 mm (from *z* = −5 mm to 5 mm). We also note that the resolution increased noticeably by introducing the novel antenna (red circles) when compared to the spatial resolution of the old antenna (black squares); in other words, the transverse resolution improved by approximately 33% compared to that of the old antenna having *R*_T_ = 1.5 mm.

Obviously, the spatial resolution will improve if we choose the high-frequency components, as shown in Figure 1f. Conversely, the TOF information is no longer available upon the spectral analysis. Most of the literature on 3D imaging has focused on the resolution in terms of the specific frequency range [31,32,48]; a direct comparison to our results is restricted here. Focusing on the TOF imaging systems, the focal spot size of 2.3 mm has been reported with the refractive lens systems [16]. In our work, we optimized the resolution in the THz-TOF systems based on the novel THz antenna. This was possible by adopting the reflective optics, which enables us to preserve the high-frequency components, particularly without sacrificing the measurement speed with the help of the galvano scanning methods.

### 3.2. Measurement of Longitudinal Resolution

**Figure 3:**
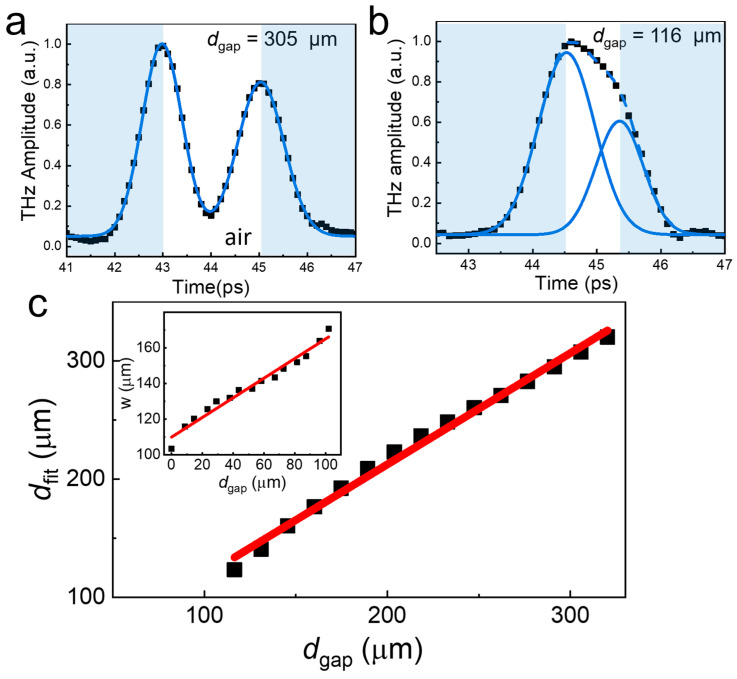
(**a**) A-scan envelope of the reflected waves for the device with an air gap (*d*_gap_) = 305 μm enclosed by two quartz substrates. The blue line is a fit to the data with a double Gaussian function. (**b**) A-scan envelope of the reflected waves with *d*_gap_ = 116 μm. (**c**) The estimated gap width (*d*_fit_) by fitting the THz reflection envelope with the double Gaussian functions as a function of *d*_gap_ as shown in (**a**,**b**) The red line is a fit to the data with a linear function. (inset). The 1/e^2^ halfwidth (*w*) of the Gaussian function was obtained by fitting the THz envelope with a single Gaussian function.

The longitudinal resolution is determined by the temporal width of the THz pulse, as shown in Figure 3, whereas the transverse resolution is determined by the imaging optics and wavelength of the THz (Figure 2). To address the longitudinal resolution, we fabricated a gap structure with various sizes, in which the gap is filled by air, enclosed by two quartz plates. Here, we varied the air gap (*d*_gap_) from 0 μm to 320 μm by tilting the top plate with respect to the bottom plate. The A-scan data for the reflected THz amplitude with *d*_gap_ = 305 μm and 116 μm are demonstrated in Figure 3a,b, respectively. The THz envelope extracted via the Hilbert transformation is characterized by a double peak for *d*_gap_ = 305 μm, with the peaks well separated from each other.

By fitting with the Gaussian function, the difference between the two peaks is found at Δ*T* = 2.03 ps, which is consistent with the measured *d*_gap_, whereas the full width at half maximum of the respective envelope is found at 1.0 ps. The envelope function was fitted well with the Gaussian function with a standard deviation of less than 1%. Conversely, a careful fitting process is required when *d*_gap_ is close to the longitudinal resolution of the system, in which we found Δ*T* = 0.77 ps for *d*_gap_ = 116 μm (Figure 3b). The fitted gap size (*d*_fit_) as a function of *d*_gap_ is plotted in Figure 3c, in which the fitted value matches well with *d*_gap_. Conversely, when *d*_gap_ < *w*, where *w* is the 1/e^2^ halfwidth of a single Gaussian function, the gap size information is no longer available from the fitting process with a double Gaussian function. Instead, it can be estimated from the broadening of the reflected pulse by obtaining *w* with a single Gaussian function (inset, Figure 3c). It can be seen that *w* increased from *w* = 116 μm of *d*_gap_ = 8.7 μm to *w* = 170 μm of *d*_gap_ = 116 μm. Consequently, the linear relationship between *w* and *d*_gap_ helps us to obtain the gap size information down to a near-zero gap size.

### 3.3. Phase of Reflected Pulses from Gap Structures with Variable Height

Importantly, the phase information obtained in nondestructive testing with the THz–TDS system allows us to address the nature of the gap enclosed by the insulating materials—that is, whether it is filled by the air or by the polymer, whose refractive index is larger than that of the enclosure. For example, it is critically important to address the types of defects in the packaged semiconducting chips, namely whether the gap is void because of delamination or it is filled by epoxy. It is very important to address this issue because reliable mechanical support for the packaged chip is essential for the physical protection of the device, distribution of electrical power, and heat dissipation of circuits [41]. This is possible with the THz-TOF imaging because the waves experience a 180° phase shift when they are reflected against materials with a relatively higher dielectric constant.

**Figure 4:**
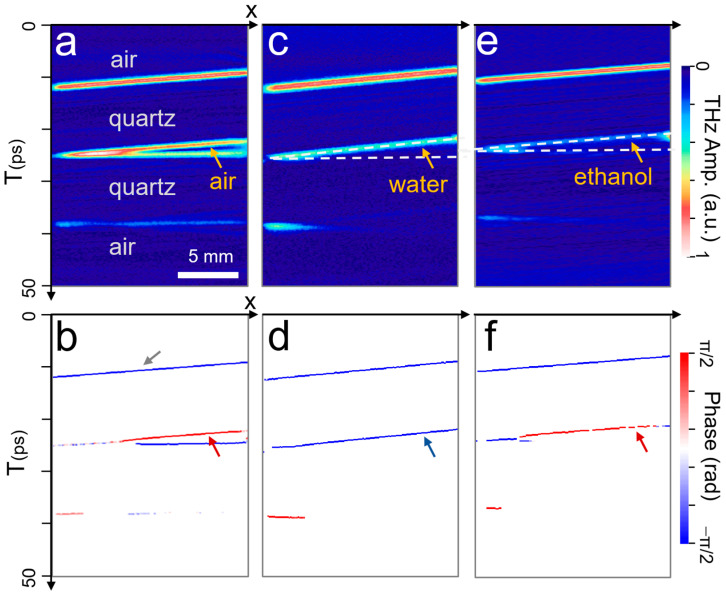
(**a**,**b**) B-scan images for the reflected amplitude (**a**) and phase (**b**) for the samples with a tilted gap that is filled with air. (**c**,**d**) B-scan images for amplitude (**c**) and phase (**d**) when the gap is filled by water solution. (**e**,**f**) B-scan images for amplitude (**e**) and phase (**f**) when the gap is filled with ethanol whose index is lower than that of quartz.

First, we show the depth profiling directly from the B-scan images for the samples consisting of two quartz substrates with an air gap in the middle (Figure 4a). The THz signal was plotted as a function of position *x* and time delay (*T*). Here, we fitted the envelope function in the time domain with a Gaussian function and plotted the peak values when they were above a threshold value for each pixel. Therefore, the position in the *T* axis denotes the peak position (which has been broadened to be three points for clarity), whereas the color scale denotes the reflection amplitude at the peak position. The THz reflection was dominated by the signal from the air–quartz and quartz–air interfaces and so there is a gap structure between the two substrates.

In Figure 4b, we show the B-scan image in terms of the phase function obtained by the Hilbert transformation. Here, the positive polarity (in-phase) indicates the reflection from the surfaces with the lower-indexed medium, whereas the negative polarity (out-of-phase) implies the reflection from the higher-indexed medium. For example, the reflection at the top surface of the quartz plate (indicated by a grey arrow) shows the negative phase because the index of the quartz is higher than that of the air. Importantly, the positive phase in the upper interface of the gap region (red arrow in Figure 4b) indicates that the gap is filled by dielectric material with the lower-indexed medium, which, in this case, is the air.

In contrast, the phase of the upper interface of the gap exhibits a negative value (Figure 4c,d), indicated by a blue arrow in (d), when the gap is filled by water (*n*_water_~2.2). This is because the index of the water is higher than that of the quartz (*n*_quartz_~1.9). We also note that the signal from the bottom interface of the gap is significantly suppressed because of the large attenuation by the water layer. We note that this is similar to the case when the gap is filled by a polymer such as epoxy which is highly absorptive in the THz frequency range [33]. Finally, we show the B-scan images for amplitude and phase (Figure 4e,f) when the gap is filled by ethanol (*n*_ethanol_~1.6), whose index is slightly smaller than that of quartz [49]. Now, the phase at the top interface of the gap shows a positive value (red arrow), as in the case of the air gap. We also note that the amplitude of the reflected waves (Figure 4e) is small compared to that of the air gap because the dielectric contrast between the quartz and ethanol is relatively low.

### 3.4. TOF Imaging of Representative Packaged Chips

**Figure 5:**
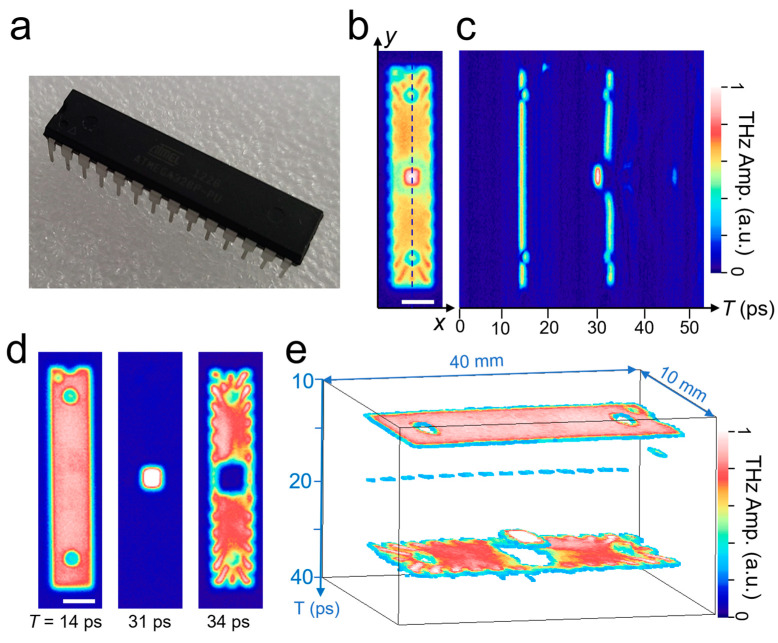
(**a**) Photograph of the chip with dimensions of 8 mm × 35 mm × 3.3 mm. (**b**) Time-integrated C-scan envelopes for the packaged device. (**c**) B-scan images along the dashed line in (**b**). Scale bar = 5 mm. (**d**) A series of C-scan (*xy*) images for T = 14, 31, and 34 ps, respectively. (**e**) Reconstructed 3D image for the device.

Figure 5 provides an example of THz-TOF imaging for a packaged chip with a relatively large thickness. We note that the ASOPS system, with a large scan range, enables NDT on objects with a large thickness. In Figure 5a, a photograph of a semiconductor package with dimensions of 8 mm × 35 mm × 3.3 mm is shown. Figure 5b shows a time-integrated C-scan image for the device, and Figure 5c is the corresponding B-scan image (as a function of *y* and *T*) along the *y*-axis at the center of the device. Figure 5d shows a series of C-scan images for different *z* locations for the sample—that is, the THz reflection images at the top, chip, and leadframe surfaces appear at *T* = 14 ps, 31 ps, and 34 ps, respectively. Considering the refractive index of the packaging material (*n* = 2.0), the TOF time differences reveal that the chip surface is located at 1.28 mm (Δ*T* = 17 ps) beneath the package top surface. Conversely, the leadframe surface appears at 1.5 mm (Δ*T* = 20 ps) below the top surface. Consequently, the THz-TOF data can then be reconstructed into 3D images (Figure 5e). Here, different colors were assigned for the reflection amplitude, with red and white colors indicating the high reflection amplitude. The chip location is clearly identified by the very large reflection amplitude, which is because semiconducting chips have a larger reflectance than insulating materials used as enclosers. In this regard, 3D mapping techniques, wrapped with their reflection amplitude, will be very effective in locating various structures embedded in the insulating materials, potentially including their material information.

### 3.5. Peak Envelopes Modulated with Phase Polarity for Defect Identification

**Figure 6:**
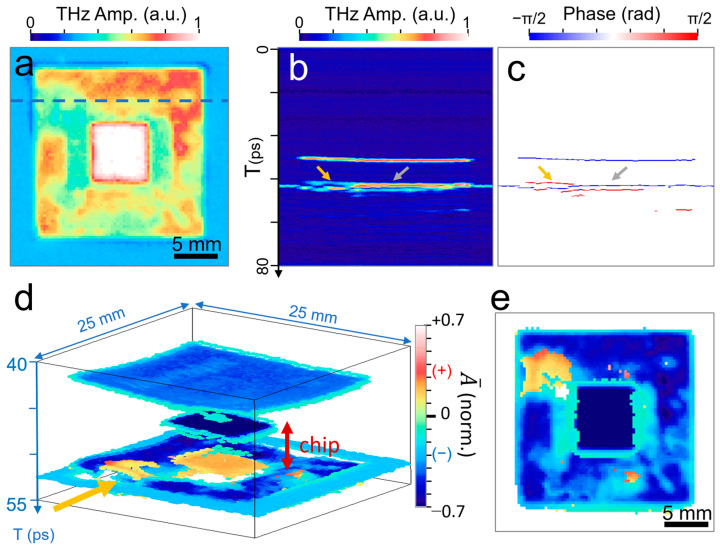
(**a**) Time-integrated C-scan image for a packaged chip with dimensions of 20 mm × 20 mm × 1 mm. (**b**) B-scan image for the reflected amplitude taken along the dashed line in (**a**). (**c**) B-scan image for the phase of the reflected waves. The orange and grey arrows indicate the void and epoxy-rich regions, respectively.(**d**) 3D image of reflected amplitudes in terms of the peak envelope modulated with phase polarity. (**e**) C-scan image for the peak envelope with phase polarity for the second peaks appearing in the time domain to highlight the delamination area (red) exclusively.

An example of identifying defects in packaged chips is illustrated in Figure 6, with a packaged device with the dimensions 20 mm × 20 mm × 1 mm. For improved image quality, we used a sample stage scan for both the *x*- and *y*-axis because the chip size is rather large. Figure 6a illustrates the time-integrated C-scan image of the device, which contains a region for the chip in the middle that is represented by a bright reflection region. In many cases, the presence of defects is not clear from the C-scan images because most of the packaged chips show similar irregular patterns outside the chip area, regardless of the presence of defects. Conversely, B-scan (*x*-*T* plot) images clearly exhibit the presence of gap structures in the middle of the packages (Figure 6b). Here, the THz amplitude is plotted along the dashed line in Figure 6a and as a function of time delay. Two different types of gap regions are clearly identified (orange and grey arrows), with gap sizes corresponding to Δ*T* = 1.9 ps and 1.7 ps, respectively. As previously discussed, the types of defects can be addressed by phase mapping (Figure 6c). In other words, it shows that the phase at the top interface exhibited a positive value (in phase) in the region denoted by an orange arrow, whereas it shows a negative value (out of phase) in the region denoted by a grey arrow. Identifying the types of defects in the packaged chips—that is, whether the gap is a void or epoxy-rich region—is our primary concern. In particular, locating the delaminated area is essential because it could cause detrimental failures to the chips in terms of mechanical stability and heat dissipation [41]. This is possible because THz waves suffer from a phase change when they are reflected at an interface that has a large dielectric constant.

The image was reconstructed as a 3D image by plotting the peak envelope modulated with phase polarity ($\bar{A}$) as a function of *x*, *y*, and *T* (Figure 6d). In other words, we plotted $\bar{A}=A_{THz}\times sign(\phi )$, where $A_{THz}$ is the normalized peak envelope and sign(ϕ) is the sign of the phase at the peak. Here, $A_{THz}$ was normalized by that of the reflection at the top surface of the chip, which has the highest reflection value. The positive (in-phase) and negative (out-of-phase) polarities again appeared, depending on the types of reflection. For example, the top surface of the package and that of the embedded chip (colored in dark blue at the center) exhibit negative polarity, whereas positive polarity is shown at the bottom of the chip (colored in orange at the center). This is consistent with the result shown in Figure 6c. More importantly, a positive phase appears in the region indicated by an arrow (orange), which clearly indicates the presence of delamination, whereas the epoxy-rich regions were not clearly identified in the 3D image. This is because the phase for the epoxy-rich region is the same as that of defect-free areas. Figure 6e shows a 2D image (C-scan), in which we plot the phase-modulated envelope for the second peaks appearing along the *T* axis. This is to exclude reflection from the top package surface and focus on the interface between the top and bottom plastic enclosures. This is the layer in which various types of defects occur during the package sealing process. As a result, both 2D and 3D imaging, based on phase-modulated peak envelopes, are shown to be unique and powerful tools for identifying the types of defects and, in particular, delamination, which is critical to chip stability.

## 4. Conclusions

We developed rapid THz-TOF imaging results consisting of reflective optics, in which a novel THz antenna based on Fe:InGaAs/InAlAs delivers a better spatial resolution and enhanced signal-to-noise ratio. We also incorporated the ASOPS technique, enabling a rapid A-scan rate of 100 Hz/scan with a large time-delay span of 100 ps. We characterized the transverse resolution with a focal length of 5 cm; the resolution in terms of the knife-edge 10−90% transition reaches 1.0 mm at the focal plane. In contrast, the longitudinal resolution reached 100 μm, which is determined by the temporal pulse width, whereas the gap width can be estimated indirectly by measuring the broadening of the pulse when the gap size is lower than the pulse width. We studied the phase shift in the reflected THz waves by using the gap structure when it is filled with air, water, and ethanol solution. The novel phase analysis revealed that we can identify the types of reflection depending on the dielectric contrast at the interface. Our 3D imaging tool was very effective in the nondestructive inspection of a packaged semiconducting chip with a high transverse and longitudinal resolution. Importantly, the phase information in 2D and 3D images is demonstrated to be powerful for identifying the delaminated region, which has a large phase contrast with opposite polarity.

## Data Availability

Not applicable.

## References

[1] Ogawa Y., Hayashi S., Oikawa M., Otani C., Kawase K. (2008). Interference terahertz label-free imaging for protein detection on a membrane. Opt. Express.

[2] Zhong S., Shen Y.C., Ho L., May R.K., Zeitler J.A., Evans M., Taday P.F., Pepper M., Rades T., Gordon K.C. (2011). Non-destructive quantification of pharmaceutical tablet coatings using terahertz pulsed imaging and optical coherence tomography. Opt. Lasers Eng..

[3] Tomaino J.L., Jameson A.D., Paul M.J., Kevek J.W., Van Der Zande A.M., Barton R.A., Choi H., McEuen P.L., Minot E.D., Lee Y.S. (2012). High-contrast imaging of graphene via time-domain terahertz spectroscopy. J. Infrared Millim. Terahertz Waves.

[4] Park S.J., Hong J.T., Choi S.J., Kim H.S., Park W.K., Han S.T., Park J.Y., Lee S., Kim D.S., Ahn Y.H. (2014). Detection of microorganisms using terahertz metamaterials. Sci. Rep..

[5] Park S.H., Jang J.W., Kim H.S. (2015). Non-destructive evaluation of the hidden voids in integrated circuit packages using terahertz time-domain spectroscopy. J. Micromechanics Microeng..

[6] Kim H.S., Ha N.Y., Park J.Y., Lee S., Kim D.S., Ahn Y.H. (2020). Phonon-Polaritons in Lead Halide Perovskite Film Hybridized with THz Metamaterials. Nano Lett..

[7] Wang L. (2021). Terahertz imaging for breast cancer detection. Sensors.

[8] Jun S.W., Ahn Y.H. (2022). Terahertz thermal curve analysis for label-free identification of pathogens. Nat. Commun..

[9] Kawase K., Ogawa Y., Watanabe Y., Inoue H. (2003). Non-destructive terahertz imaging of illicit drugs using spectral fingerprints. Opt. Express.

[10] Federici J.F., Schulkin B., Huang F., Gary D., Barat R., Oliveira F., Zimdars D. (2005). THz imaging and sensing for security applications—Explosives, weapons and drugs. Semicond. Sci. Technol..

[11] Zhong H., Xu J., Xie X., Yuan T., Reightler R., Madaras E., Zhang X.C. (2005). Nondestructive defect identification with terahertz time-of-flight tomography. IEEE Sens. J..

[12] Karpowicz N., Redo A., Zhong H., Li X., Xu J., Zhang X.C. Continuous-wave terahertz imaging for non-destructive testing applications. Proceedings of the Joint 30th International Conference on Infrared and Millimeter Waves and 13th International Conference on Terahertz Electronics, IRMMW-THz 2005.

[13] Shen Y.C., Lo T., Taday P.F., Cole B.E., Tribe W.R., Kemp M.C. (2005). Detection and identification of explosives using terahertz pulsed spectroscopic imaging. Appl. Phys. Lett..

[14] Schirmer M., Fujio M., Minami M., Miura J., Araki T., Yasui T. (2010). Biomedical applications of a real-time terahertz color scanner. Biomed. Opt. Express.

[15] Kawase K., Shibuya T., Hayashi S., Suizu K. (2010). THz imaging techniques for nondestructive inspections. Comptes Rendus Phys..

[16] Jin K.H., Kim Y.G., Cho S.H., Ye J.C., Yee D.S. (2012). High-speed terahertz reflection three-dimensional imaging for nondestructive evaluation. Opt. Express.

[17] Fan S., Li T., Zhou J., Liu X., Liu X., Qi H., Mu Z. (2017). Terahertz non-destructive imaging of cracks and cracking in structures of cement-based materials. AIP Adv..

[18] Ahi K., Shahbazmohamadi S., Asadizanjani N. (2018). Quality control and authentication of packaged integrated circuits using enhanced-spatial-resolution terahertz time-domain spectroscopy and imaging. Opt. Lasers Eng..

[19] Fuse N., Sugae K. (2019). Non-destructive terahertz imaging of alkali products in coated steels with cathodic disbanding. Prog. Org. Coat..

[20] Zhang J.Y., Ren J.J., Li L.J., Gu J., Zhang D.D. (2020). THz imaging technique for nondestructive analysis of debonding defects in ceramic matrix composites based on multiple echoes and feature fusion. Opt. Express.

[21] Karpowicz N., Zhong H., Zhang C., Lin K.I., Hwang J.S., Xu J., Zhang X.C. (2005). Compact continuous-wave subterahertz system for inspection applications. Appl. Phys. Lett..

[22] Karpowicz N., Zhong H., Xu J., Lin K.I., Hwang J.S., Zhang X.C. (2005). Comparison between pulsed terahertz time-domain imaging and continuous wave terahertz imaging. Semicond. Sci. Technol..

[23] Lien Nguyen K., Johns M.L., Gladden L.F., Worrall C.H., Alexander P., Beere H.E., Pepper M., Ritchie D.A., Alton J., Barbieri S. (2006). Three-dimensional imaging with a terahertz quantum cascade laser. Opt. Express.

[24] Kim J.-Y., Song H.-J., Yaita M., Hirata A., Ajito K. (2014). CW-THz vector spectroscopy and imaging system based on 1.55-µm fiber-optics. Opt. Express.

[25] Lee I.S., Lee J.W. (2020). Nondestructive internal defect detection using a CW-THz imaging system in XLPE for power cable insulation. Appl. Sci..

[26] Mathanker S.K., Weckler P.R., Wang N. (2013). Terahertz (THz) applications in food and agriculture: A review. Trans. ASABE.

[27] Ok G., Park K., Kim H.J., Chun H.S., Choi S.W. (2014). High-speed terahertz imaging toward food quality inspection. Appl. Opt..

[28] Wang K., Sun D.W., Pu H. (2017). Emerging non-destructive terahertz spectroscopic imaging technique: Principle and applications in the agri-food industry. Trends Food Sci. Technol..

[29] Afsah-Hejri L., Hajeb P., Ara P., Ehsani R.J. (2019). A Comprehensive Review on Food Applications of Terahertz Spectroscopy and Imaging. Compr. Rev. Food Sci. Food Saf..

[30] Nagatsuma T., Nishii H., Ikeo T. (2014). Terahertz imaging based on optical coherence tomography [invited]. Photonics Res..

[31] Cristofani E., Friederich F., Wohnsiedler S., Matheis C., Jonuscheit J., Vandewal M., Beigang R. (2014). Nondestructive testing potential evaluation of a terahertz frequency-modulated continuous-wave imager for composite materials inspection. Opt. Eng..

[32] Yahng J.S., Park C.S., Lee H.D., Kim C.S., Yee D.S. (2016). High-speed frequency-domain terahertz coherence tomography. Opt. Express.

[33] Yim J.H., Kim S.Y., Kim Y., Cho S., Kim J., Ahn Y.H. (2021). Rapid 3d-imaging of semiconductor chips using thz time-of-flight technique. Appl. Sci..

[34] Kim H.S., Baik S.Y., Lee J.W., Kim J., Ahn Y.H. (2021). Nondestructive tomographic imaging of rust with rapid thz time-domain spectroscopy. Appl. Sci..

[35] Hochrein T., Wilk R., Mei M., Holzwarth R., Krumbholz N., Koch M. (2010). Optical sampling by laser cavity tuning. Opt. Express.

[36] Wilk R., Hochrein T., Koch M., Mei M., Holzwarth R. (2011). OSCAT: Novel technique for time-resolved experiments without moveable optical delay lines. J. Infrared Millim. Terahertz Waves.

[37] Yasui T., Saneyoshi E., Araki T. (2005). Asynchronous optical sampling terahertz time-domain spectroscopy for ultrahigh spectral resolution and rapid data acquisition. Appl. Phys. Lett..

[38] Bartels A., Thoma A., Janke C., Dekorsy T., Dreyhaupt A., Winnerl S., Helm M. (2006). High-resolution THz spectrometer with kHz scan rates. Opt. Express.

[39] Kim Y., Yee D.-S. (2010). High-speed terahertz time-domain spectroscopy based on electronically controlled optical sampling. Opt. Lett..

[40] Pałka N., Maciejewski M., Kamiński K., Piszczek M., Zagrajek P., Czerwińska E., Walczakowski M., Dragan K., Synaszko P., Świderski W. (2022). Fast THz-TDS Reflection Imaging with ECOPS—Point-by-Point versus Line-by-Line Scanning. Sensors.

[41] Yahyapour M., Jahn A., Dutzi K., Puppe T., Leisching P., Schmauss B., Vieweg N., Deninger A. (2019). Fastest Thickness Measurements with a Terahertz Time-Domain System Based on Electronically Controlled Optical Sampling. Appl. Sci..

[42] Globisch B., Dietz R.J.B., Kohlhaas R.B., Göbel T., Schell M., Alcer D., Semtsiv M., Masselink W.T. (2017). Iron doped InGaAs: Competitive THz emitters and detectors fabricated from the same photoconductor. J. Appl. Phys..

[43] Kocic N., Wichmann M., Hochrein T., Heidemeyer P., Kretschmer K., Radovanovic I., Mondol A.S., Koch M., Bastian M. (2014). Lenses for terahertz applications: Development of new materials and production processes. AIP Conf. Proc..

[44] Harris Z.B., Virk A., Khani M.E., Arbab M.H. (2020). Terahertz time-domain spectral imaging using telecentric beam steering and an f-θ scanning lens: Distortion compensation and determination of resolution limits. Opt. Express.

[45] Kong D.Y., Wu X.J., Wang B., Gao Y., Dai J., Wang L., Ruan C.J., Miao J.G. (2018). High resolution continuous wave terahertz spectroscopy on solid-state samples with coherent detection. Opt. Express.

[46] Wachulak P.W., Torrisi A., Bartnik A., Adjei D., Kostecki J., Wegrzynski L., Jarocki R., Szczurek M., Fiedorowicz H. (2015). Desktop water window microscope using a double-stream gas puff target source. Appl. Phys. B.

[47] Wachulak P.W., Torrisi A., Bartnik A., Węgrzyński Ł., Fok T., Fiedorowicz H. (2016). A desktop extreme ultraviolet microscope based on a compact laser-plasma light source. Appl. Phys. B.

[48] Di Fabrizio M., D’Arco A., Mou S., Palumbo L., Petrarca M., Lupi S. (2021). Performance Evaluation of a THz Pulsed Imaging System: Point Spread Function, Broadband THz Beam Visualization and Image Reconstruction. Appl. Sci..

[49] Park S.J., Yoon S.A.N., Ahn Y.H. (2016). Dielectric constant measurements of thin films and liquids using terahertz metamaterials. RSC Adv..

